# Highly Stable Passively Q-Switched Erbium-Doped All-Fiber Laser Based on Niobium Diselenide Saturable Absorber

**DOI:** 10.3390/molecules26144303

**Published:** 2021-07-16

**Authors:** Ping Hu, Jiajia Mao, Hongkun Nie, Ruihua Wang, Baitao Zhang, Tao Li, Jingliang He, Kejian Yang

**Affiliations:** 1 Institute of Novel Semiconductors, State Key Laboratory of Crystal Materials, Shandong University, Jinan 250100, China; 202020452@mail.sdu.edu.cn (P.H.); 201912560@mail.sdu.edu.cn (J.M.); hknie@sdu.edu.cn (H.N.); rhwang@sdu.edu.cn (R.W.); btzhang@sdu.edu.cn (B.Z.); litao@sdu.edu.cn (T.L.); jlhe@sdu.edu.cn (J.H.); 2 China Key Laboratory of Laser & Infrared System, Ministry of Education Shandong University, Qingdao 266237, China; 3 Collaborative Innovation Center of Light Manipulations and Applications, Shandong Normal University, Jinan 250358, China; 4 Shenzhen Research Institute, Shandong University, Shenzhen 518057, China; 5 State Key Laboratory of Quantum Optics and Quantum Optics Devices, Shanxi University, Taiyuan 030006, China

**Keywords:** niobium diselenide, fiber laser, Q-switched, saturable absorber

## Abstract

A saturable absorber (SA) based on niobium diselenide (NbSe_2_), which is a layered transition metal dichalcogenide (TMD) in the VB group, is fabricated by the optically driven deposition method, and the related nonlinear optical properties are characterized. The modulation depth, saturable intensity, and nonsaturable loss of the as-prepared NbSe_2_ nanosheet-based SA are measured to be 16.2%, 0.76 MW/cm^2^, and 14%, respectively. By using the as-fabricated NbSe_2_ SA, a highly stable, passively Q-switched, erbium-doped, all-fiber laser is realized. The obtained shortest pulse width is 1.49 μs, with a pulse energy of 48.33 nJ at a center wavelength of 1560.38 nm. As far as we know, this is the shortest pulse duration ever obtained by an NbSe_2_ SA in a Q-switched fiber laser.

## 1. Introduction

Short-pulsed, all-fiber lasers have been widely used in various fields such as material processing, medical diagnosis, industrial processing, light detection, and ranging (LIDAR, also LADER), optical fiber communication, and spectroscopy [1,2,3]. The emergence and development of Q-switching technology is an important breakthrough in the history of laser development [4]. It compresses the laser energy into extremely narrow pulses for emission, so that the peak power of the light source can be increased by several orders of magnitude. The Q-switching mechanism mainly involves the generation of short pulses, whose pulse duration can reach the order of microseconds (μs), nanoseconds (ns), and even picoseconds (ps), while the repetition frequency can range from several Hz to hundreds of kilohertz (kHz) [5,6]. Passive Q-switching technology based on SAs has been a popular way to achieve pulsed lasers due to its advantages of compactness, high efficiency, and low cost [7,8,9]. In recent years, with the development of nonlinear optical materials, two-dimensional (2D) materials have received widespread attention due to their novel electronic and optical performance [10,11,12,13]. Despite the thin atomic layer, many 2D materials interact strongly with light in a broad spectral band, which is helpful for optical modulation. However, the nonlinear optical response characteristics of the 2D materials inevitably affect the repeatability and reliability of pulsed fiber lasers [14]. Therefore, exploring suitable materials with excellent nonlinear optical response properties is still the key to the development of high-performance pulsed fiber lasers. Among the layered 2D materials, TMDs have been widely studied due to their excellent nonlinear optical performance introduced by the high third-order nonlinear susceptibility [15]. Compared with other typical 2D materials, such as graphene and black phosphorous (BP), the band gap of TMDs increases with the decrease in the number of layers [16], which introduces broadband absorption helpful for optical modulation [17]. In addition, TMDs have high carrier mobility [18], which makes them possess fast nonlinear response to incident light, thus making them suitable for generating narrow pulses.

Among the TMD materials, extensive research has been conducted on materials with semiconductor properties such as WS_2_, MoS_2_, WSe_2_, and MoSe_2_ [19,20,21,22], which show promising performance in generating pulsed laser pulses. Compared with the TMD materials with semiconductor properties, 2D nanostructured TMDs with metal layers, such as NbS_2_ and NbSe_2_, exhibit lower fabrication cost, stronger optical absorption, and higher carrier mobility [23,24,25,26]. Recently, NbSe_2_ has been clearly proven to have good saturable absorption properties in the spectral range from 1 to 1.5 μm, due to ultra-fast saturation recovery time and wide absorption band [27,28,29,30]. Furthermore, NbSe_2_ has excellent environmental stability, so it is very helpful for generating long-term stable laser pulses, especially in fiber lasers. In 2018, Shi et al. prepared an NbSe_2_ quantum dot (QD)-based SA with modulation depth and saturable absorption intensity of 3.72% and 3.155 GW/cm^2^, respectively, and employed it to achieve a Q-switching fiber laser, which delivered pulses with a duration of 2.53 μs and single pulse energy of 98.19 nJ at a center wavelength of 1533 nm [27,28]. The SA was fabricated by dropping NbSe_2_ QDs solution onto the side-projection face of a D-shaped fiber, which could improve both the damage threshold and thermal stability of the SA. Although NbSe_2_ QDs and D-shaped fiber have stronger evanescent field interactions than the ordinary single-mode fiber, greater coupling loss would also be introduced during the fusion splicing process with single-mode fiber. In addition, the special structure of the SA based on D-shaped fiber also determines relatively low modulation depth and high saturable absorption intensity, which is harmful for achieving a low-threshold Q-switched laser with narrow pulse duration. 

In this paper, a kind of NbSe_2_ nanosheet SA is fabricated by the optically driven deposition method, and the nonlinear saturable absorption properties are characterized. The NbSe_2_ nanosheet-based SA shows a large modulation depth of 16.2%, and a low saturation intensity of 0.76 MW/cm^2^, which provides the possibility to achieve narrow laser pulses with low threshold. By employing the as-prepared NbSe_2_ SA in an all-fiber, erbium-doped fiber laser, a stable passive Q-switching operation is realized. The obtained pulse repetition rate ranges from 15.12 to 64.14 kHz within the pump power range. The shortest pulse duration is 1.49 μs, corresponding to a single pulse energy of 48.33 nJ. As far as we know, this is the shortest pulse duration ever obtained by an NbSe_2_ SA in a Q-switched fiber laser so far. The results clearly show that such a kind of NbSe_2_ SA has great potential for achieving all-fiber pulsed laser operation.

## 2. Preparation and Characterization of NbSe_2_

### 2.1. Preparation of NbSe_2_

**Figure 1:**
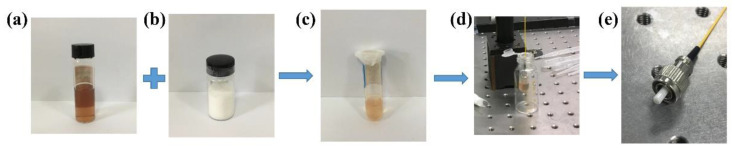
Preparation process of NbSe_2_-PVA SA. (**a**) NbSe_2_ powder; (**b**) PVA powder; (**c**) NbSe_2_-PVA composite dispersion; (**d**) optically driven deposition setup; (**e**) NbSe_2_ nanosheet-coated fiber connector.

The NbSe_2_ nanosheet SA is prepared by the optically driven deposition method due to its simplicity and high efficiency. The preparation process is shown in Figure 1. The NbSe_2_ nanosheet powder was purchased from Shenzhen HAOLAI Technology Co., Ltd., Shenzhen, China. In the preparation method, 10 mg of NbSe_2_ powder and 10 mL ethanol are first mixed to form the dispersion of NbSe_2_ nanosheets. The dispersion is fully stirred for 15 min and subjected to ultrasonication for 2 h. After that, the dispersion is centrifuged at 3000 rpm for 30 min, until the NbSe_2_ nanosheets are completely dispersed. Then, 280 mg of polyvinyl acetate (PVA) powder is added into 7 mL of NbSe_2_ nanosheet dispersion, and sonicated for 30 min to prepare a uniform NbSe_2_-PVA composite with embedded nanosheets. In this special polymer matrix, PVA can prevent oxidation while forming a NbSe_2_ nanosheet film. Next, the end face of an optical fiber connector is immersed into the prepared NbSe_2_-PVA composite solution. Then, the coated fiber is connected to a laser (976 nm, 25 mW) for 20 min. Finally, the coated fiber is disconnected and allowed to dry for 24 h at room temperature.

### 2.2. Characterization of NbSe_2_ SA Properties

**Figure 2:**
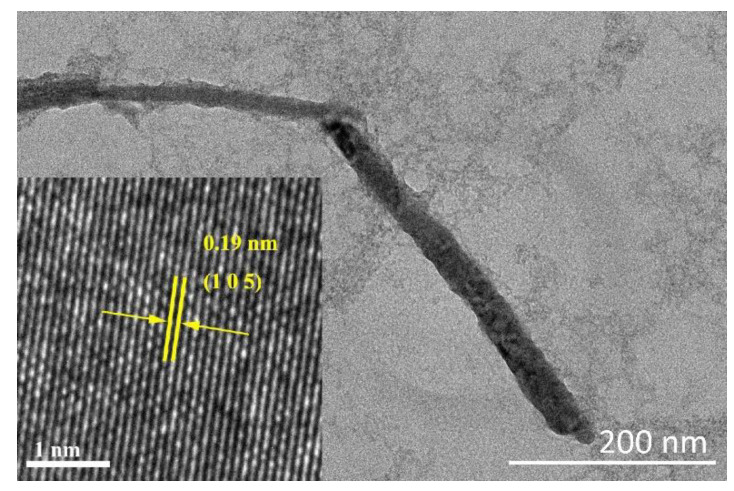
TEM image of NbSe_2_ nanosheets. Inset: HRTEM image of NbSe_2_ nanosheets.

The microstructure and thickness of NbSe_2_ nanosheets are characterized by a transmission electron microscope (TEM) (JEOL, JEM 2100F), which is driven at an accelerating voltage of 200 kV. A TEM image of the NbSe_2_ nanosheet SA with a resolution of 200 nm is shown in Figure 2. It can be seen that the NbSe_2_ nanosheets demonstrate clear layer structure and rod shape. The inset is a high-resolution TEM (HRTEM) image of the NbSe_2_ nanosheets, from which no obvious defects are found, clearly proving the excellent structure of the NbSe_2_ nanosheets. The lattice distance is about 0.19 nm, which corresponds to the (1, 0, 5) lattice plane.

**Figure 3:**
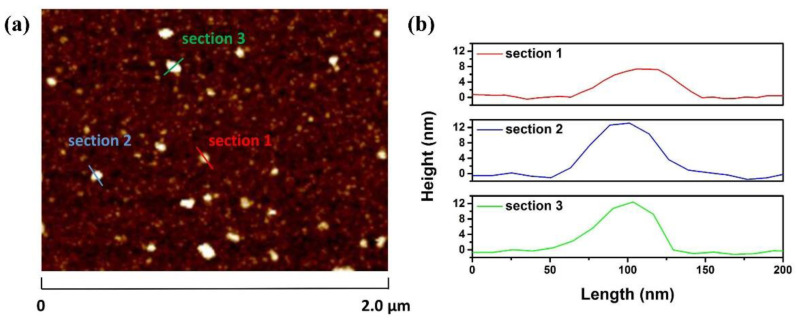
(**a**) AFM image of NbSe_2_ SA; (**b**) height profiles of NbSe_2_ SA.

In order to confirm the thickness of NbSe_2_ nanosheets in NbSe_2_-PVA film, atomic force microscopy (AFM) (Dimension Icon, Veeco Instruments Inc., Plainview, NY, USA) measurement is performed. The NbSe_2_-PVA composite dispersion is deposited onto a quartz substrate by the spin coating method, and dried to form a NbSe_2_-PVA film. To avoid affecting the number of NbSe_2_ nanosheet layers, the prepared sample is placed in ethanol solvent and sonicated for 10 min to remove excessive PVA on the surface. Subsequently, the sample is dried for characterization. The corresponding AFM images and height profiles are shown in Figure 3a,b. Three section parts in the scope of Figure 3a are selected to characterize the heights, denoted as section 1, section 2, and section 3, respectively. It can be seen from Figure 3b that the thicknesses of the selected NbSe_2_ nanosheet parts range from 7 to 14 nm. Considering a single-layer NbSe_2_ nanosheet thickness of about 1.1 nm, the prepared NbSe_2_ SA nanosheets are determined to be about 6–13 layers [31]. The few-layered nature of the NbSe_2_ SA nanosheets makes them suitable for working at a wavelength of around 1550 nm.

**Figure 4:**
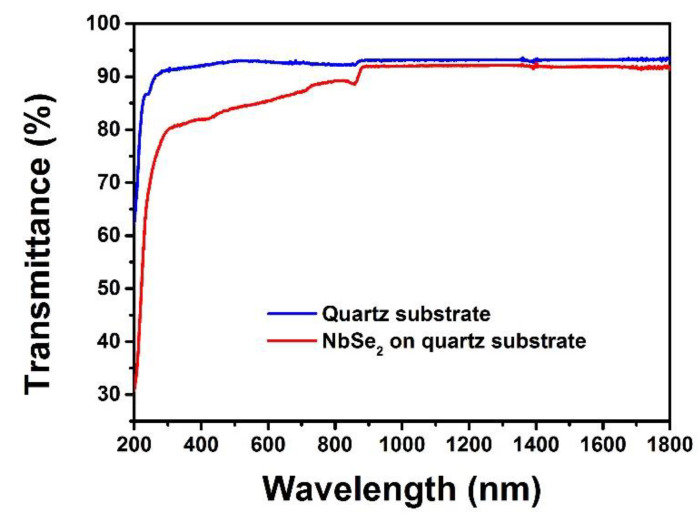
Transmittance of the quartz substrate and NbSe_2_ based on quartz substrate.

A UV/VIS/NIR spectrophotometer (U-3500, Hitachi, Japan) is further used to measure the linear transmission spectrum of NbSe_2_ nanosheet SA in a spectral range of 200–1800 nm. As shown in Figure 4, the blue line represents the transmittance of the blank quartz substrate, and the red line represents the transmittance of the NbSe_2_ nanosheet SA on the quartz substrate under the same conditions. In the near-infrared spectral range from 900 to 1800 nm, the transmittances of the quartz substrate with the NbSe_2_ nanosheet SA and the blank quartz substrate are found to be 87.5 ± 5.5% and 92.7 ± 0.5%, respectively, which shows that the NbSe_2_ nanosheet SA introduces an absorbance of about 5.2%, indicating the broad absorption properties of NbSe_2_ SA.

**Figure 5:**
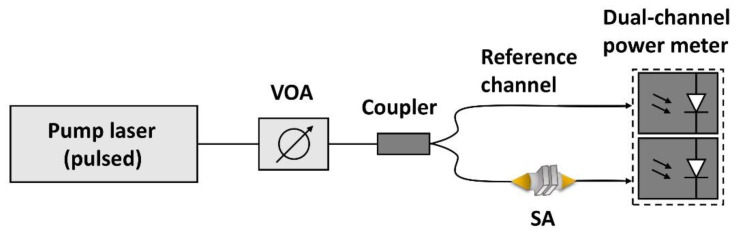
Diagram of experimental setup for balanced dual-detector measurement method.

**Figure 6:**
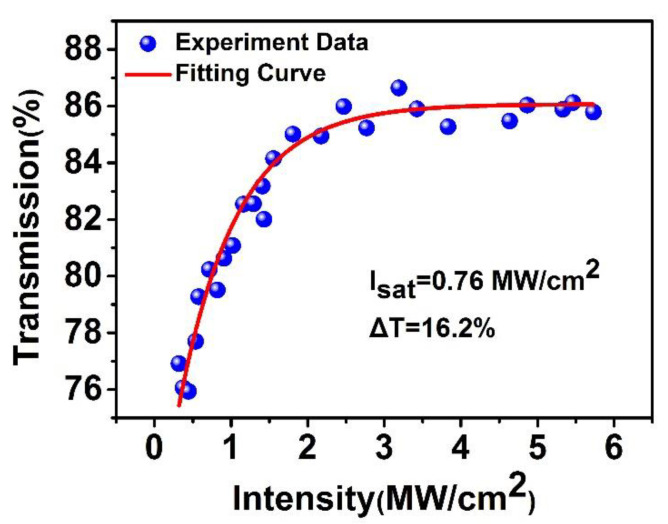
The nonlinear optical absorption properties of the NbSe_2_ SA.

The nonlinear saturable absorption properties of NbSe_2_ SA are studied by the balanced dual-detector measurement method, as shown in Figure 5. A femtosecond/picosecond laser is used as a pumping source. The working wavelength of the fiber laser used is 1550 nm, and the fundamental frequency is 10 Hz. The power is changed using a variable optical attenuator (VOA). A typical fiber coupler (50:50) splits the power between the reference and measurement arm. Then, by comparing the values of the two power meters, the transmittance curve of the sample to the laser pulse at different optical powers can be drawn. In the end, the nonlinear, power-dependent, normalized saturation absorption curve based on NbSe_2_ SA is shown in Figure 6. It can be seen from the figure that the blue dots represent experimental data. The red line in the figure shows the curve of the saturated absorption formula [32]:

T(I) = 1 − *Tns* − ∆*T* × exp(−*I*/*Isat*) (1)

where T(I) is the measured transmittance, *Tns* is the nonsaturable absorbance, Δ*T* is modulation depth, *I* is input laser intensity, and *Isat* is saturation intensity. By simulating the measured data with Equation (1), the modulation depth, saturation intensity, and nonsaturable loss are 16.2%, 0.76 MW/cm^2^, and 14%, respectively. Compared with the results reported in previous work [33,34], the NbSe_2_ SA has a higher modulation depth and a lower saturation intensity in this work. For the D-shaped fiber-based SA, its saturable absorption arises from the interaction between the evanescent field outside the D-shaped fiber and SA, introducing a large unsaturable loss due to the large interaction area. Here, due to the direct light–matter interaction between the NbSe_2_ and light at the end face of SMF, low unsaturable loss, large modulation depth, and low saturation intensity are easily obtained. The large modulation depth would help generate short pulses [35], while the low saturable intensity makes low-threshold and high-repetition rate Q-switching operation expected. As can be seen from Figure 6, when the laser power gradually increases, the optical transmittance increases to approximately 86% and remains saturated. The NbSe_2_ nanosheet sample exhibits a typical saturated absorption curve.

## 3. Experimental Results of Optical Modulation and Discussions

**Figure 7:**
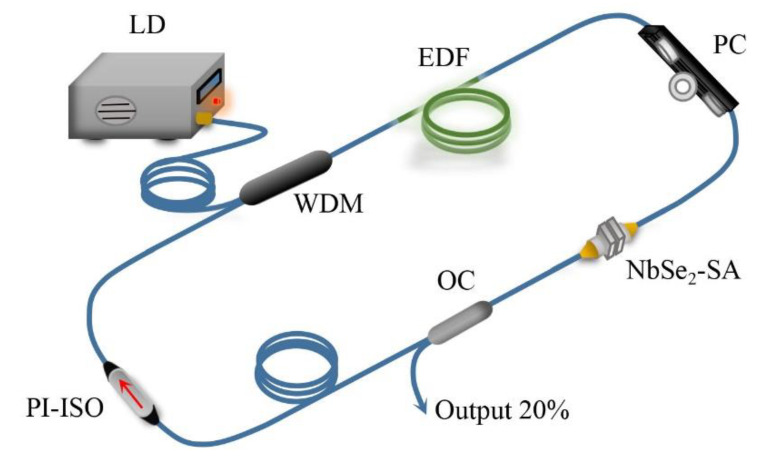
Schematic diagram of the NbSe_2_ SA passively Q-switched EDF laser experiment.

The experimental setup of the passively Q-switched, erbium-doped fiber laser based on the as-prepared NbSe_2_ SA is shown in Figure 7. The ring laser cavity includes pump source, 980/1550 nm wavelength division multiplexer (WDM), polarization controller (PC), 20/80 output coupler (OC), polarization independent isolator (PI-ISO), erbium-doped fiber (EDF), NbSe_2_ SA, and single-mode fiber (SMF). Among them, the pump source is a 976 nm laser diode (LD) with a maximum power of 515 mW. The pump light is coupled into the laser cavity through the 980/1550 nm WDM. A 6 m single mode erbium-doped fiber (Nufern-EDFC-980-HP) is used as the gain medium. The PI-ISO is used to ensure the unidirectional light propagation in the laser cavity. Since the cavity is composed of standard SMFs, a PC is required to adjust the polarization state. In addition, an OC with a coupling rate of 20% is employed to output the laser pulses. The total length of the ring laser cavity is about 11 m. The spectral and temporal properties of the output pulses are characterized by a spectrum analyzer (YOKOGAWA AQ6370B) and a digital oscilloscope (Tektronix DP04104), respectively. The average output power is measured by a power meter (Molectron PM3).

During the experiment, the PC is adjusted to change the polarization state of the cavity mode while increasing the pump power, and then the laser output pulses are observed using the spectrum analyzer and digital oscilloscope. When the pump power is increased to 41 mW, the laser begins to work in the Q-switching regime. In order to verify the function of the NbSe_2_ SA in the Q-switching, erbium-doped fiber laser, the SA is taken out of the cavity and the cavity length is kept unchanged. By adjusting the PC and pump power carefully, no Q-switching phenomenon is observed. The results affirm that the as-prepared NbSe_2_ SA is mainly responsible for the Q-switching operation. 

**Figure 8:**
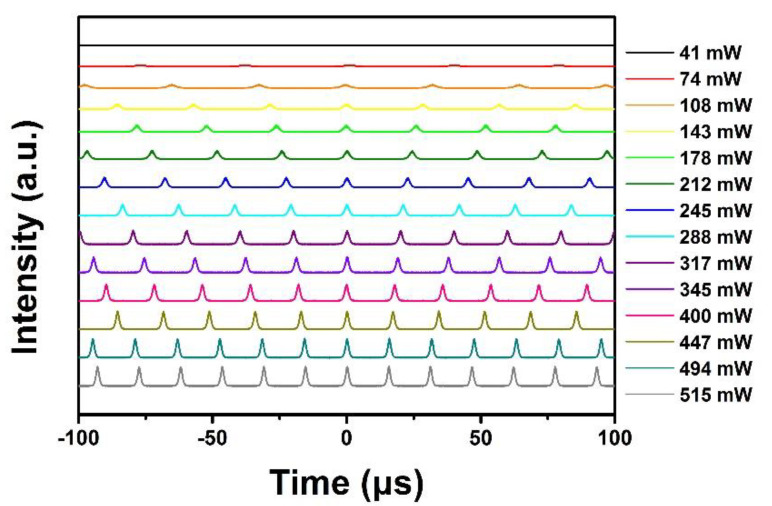
Temporal pulse sequences under different pump powers.

Figure 8 presents the output pulse sequences at different pump powers. The pulses have identical amplitudes even under a pump power above 500 mW, indicating stable Q-switching operations under different pump powers. When the pump power is increased to the maximum of 515 mW, the Q-switched pulses still remain highly stable. Due to the limitation of the pump power available in our laboratory, it is impossible to measure the affordable pump power limit of the NbSe_2_ SA; however, no damage is observed within the pump power range. In addition, the NbSe_2_ nanosheet SA exhibits high long-term stability and oxidation resistance under environmental conditions, which makes the laser remain in stable operation for more than one month, and makes it quite suitable for application in practical optical devices.

**Figure 9:**
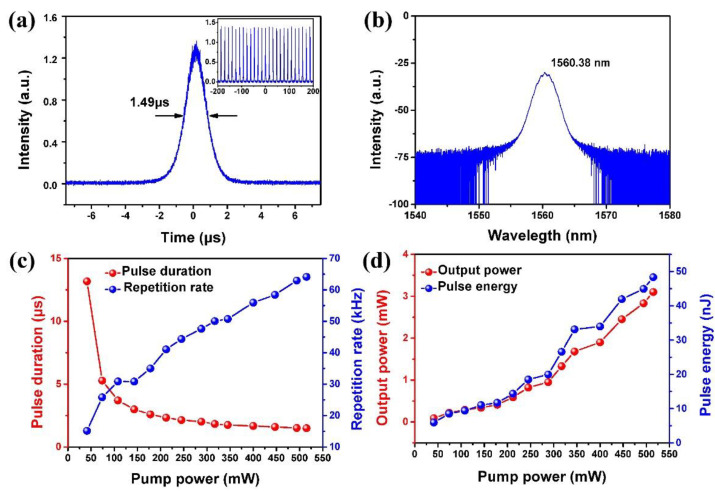
The output pulse characteristics of the NbSe_2_ SA, passively Q-switched fiber laser. (**a**) Temporal pulse shape recorded under the pump power of 515 mW, and the inset is the corresponding pulse sequence; (**b**) laser spectrum recorded under the maximum pump power; (**c**) pulse durations and pulse repetition rates versus the pump powers; (**d**) output powers and pulse energies versus the pump powers.

Figure 9a presents the temporal pulse shape with the shortest pulse width of 1.49 μs recorded under the maximum pump power of 515 mW, corresponding to a repetition rate of 64.14 kHz. Figure 9b shows the spectrum of the output pulse with a center wavelength at 1560.38 nm. The recorded pulse durations and repetition rates under different pump powers are shown in Figure 9c. When the pump power is increased from 41 to 515 mW, the pulse duration decreases from 13.18 to 1.49 μs, while the repetition rate increases from 15.12 to 64.14 kHz. Figure 9d depicts the dependence of output power and single pulse energy on the pump power. Under the maximum pump power of 515 mW, a maximum average output power of 3.1 mW is obtained with a pulse energy of 48.33 nJ, corresponding to an optical–optical conversion efficiency and slope efficiency of 0.6% and 0.64%, respectively. The slope efficiency is relatively low due to the high insertion loss of SA, but it is possible to further improve it by optimizing the unsaturated loss of NbSe_2_ SA and splitting ratio of OC. 

**Figure 10:**
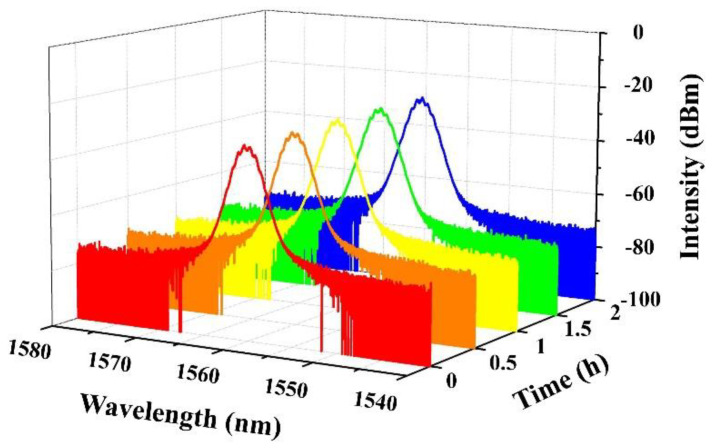
Long-term performance of the NbSe_2_ SA Q-switched fiber laser within 2 h with an interval of 30 min.

In order to test the long-term stability of the Q-switched fiber laser based on NbSe_2_ SA, the output spectrum under the maximum pump power is continuously monitored for more than 2 h with intervals of 30 min. As shown in Figure 10, the output spectrum does not change within 2 h, which proves that the Q-switched, erbium-doped fiber laser has excellent stability at room temperature. The results clearly indicate the as-prepared NbSe_2_ SA meets the requirements of long-term and stable laser operation under atmospheric conditions, which is very suitable for a nonlinear photonic device to generate narrow output pulses in fiber lasers.

For comparison, the performance of passively Q-switched, erbium-doped fiber lasers using different nanomaterial SAs is summarized in Table 1. Compared with other SAs, the NbSe_2_-based SA generates the shortest pulse duration in this work. It is indicated that the optically driven deposition method is helpful to reduce the unexpected splicing loss of the prepared NbSe_2_ SA while retaining high modulation depth and low saturation intensity, which is beneficial for generating short pulses with low pump thresholds. 

## 4. Conclusions

In conclusion, a high long-term stability, passively Q-switched, erbium-doped all-fiber laser is realized by using a few-layered NbSe_2_ SA fabricated by the optically driven deposition method. The as-prepared NbSe_2_ nanosheet SA shows good homogeneity and uniform thickness. The nonlinear optical properties of the NbSe_2_ SA are characterized in the 1550 nm spectral region, and a large modulation depth of 12.6% and a low saturation intensity of 0.76 MW/cm^2^ are demonstrated. By using the as-prepared NbSe_2_ SA, highly stable Q-switching operation is realized in the erbium-doped all-fiber laser. When the pump power is 515 mW, a maximum average output power of 3.1 mW is obtained, corresponding to a pulse energy of 48.33 nJ and a pulse duration of 1.49 μs. This is the shortest pulse duration ever achieved using an NbSe_2_ SA in a passively Q-switched fiber laser, to the best of our knowledge. The results indicate that the NbSe_2_ SA is a promising practical photonic device for all-fiber pulsed lasers. 

## Figures and Tables

**Table 1:** Comparison of passively Q-switched, Er-doped fiber lasers based on different SAs.

SA	Wavelength (nm)	Pulse Width (μs)	Pulse Energy (nJ)	Max Output Power (mW)	Refs.
graphene	1539.6	3.89	28.7	3.38	[33]
MoS_2_	1560	3.97	131.52	6	[34]
WS_2_	1560	3.71	126.96	4.82	[34]
ReS_2_	1532	2.57	38	2.48	[36]
BP	1564.16	2.98	283.91	8.55	[37]
antimonene	1559.63	1.58	37.9	2.85	[38]
NbSe_2_ QD	1533	2.53	98.19	2.00	[28]
NbSe_2_	1560.38	1.49	48.33	3.1	This work

## Data Availability

Data are contained within this article.

## Sample Availability

Samples of the compounds are not available from the authors.
